# OVsignGenes: A Gene Expression-Based Neural Network Model Estimated Molecular Subtype of High-Grade Serous Ovarian Carcinoma

**DOI:** 10.3390/cancers16233951

**Published:** 2024-11-25

**Authors:** Anastasiya Kobelyatskaya, Anna Tregubova, Andrea Palicelli, Alina Badlaeva, Aleksandra Asaturova

**Affiliations:** 1 Engelhardt Institute of Molecular Biology, Russian Academy of Sciences, 119991 Moscow, Russia; 2 National Medical Research Center for Obstetrics, Gynecology and Perinatology Named After Academician V.I. Kulakov of the Ministry of Health of Russia, 117513 Moscow, Russia; annyupitrue@mail.ru (A.T.); alinamagnaeva03@gmail.com (A.B.); a.asaturova@gmail.com (A.A.); 3 Pathology Unit, Azienda USL—IRCCS di Reggio Emilia, 42123 Reggio Emilia, Italy; andreapalicelli@hotmail.it

**Keywords:** ovarian cancer, HGSC, molecular subtype, gene expression, pathways, neural network, prediction

## Abstract

Background/Objectives: High-grade serous carcinomas (HGSCs) are highly heterogeneous tumors, both among patients and within a single tumor. Differences in molecular mechanisms significantly describe this heterogeneity. Four molecular subtypes have been previously described by the Cancer Genome Atlas Consortium: differentiated, immunoreactive, mesenchymal, and proliferative. These subtypes may have varying degrees of progression, relapse-free survival, and overall survival, as well as response to therapy. The precise determination of these subtypes is certainly necessary both for diagnosis and future development of targeted therapies within personalized medicine. Methods: In this study, we analyzed gene expression data based on bulk RNA-seq, scRNA-seq, and spatial transcriptomic data from six cohorts (totaling 535 samples, including 60 single-cell samples). Differential expression analysis was performed using the edgeR package. The KEGG database and GSVA package were used for pathways enrichment analysis. As a predictive model, a deep neural network was created using the keras and tensorflow libraries. Results: We identified 357 differentially expressed genes among the four subtypes: 96 differentiated, 33 immunoreactive, 91 mesenchymal, and 137 proliferative. Based on these, we created OVsignGenes, a neural network model resistant to the effects of platform (test dataset AUC = 0.969). We then ran data from five more cohorts through our model, including scRNA-seq and spatial transcriptomics. Conclusions: Because the differentiated subtype is located at the intersection of the other three subtypes based on PCA and does not have a unique profile of differentially expressed genes or enriched pathways, it can be considered an initiating subtype of tumor that will develop into one of the three other subtypes.

## Simple Summary

High-grade serous ovarian cancer (HGSC) has a high heterogeneity both among patients and within a single tumor. Four molecular subtypes of HGSC were previously described. These studies were based on analysis of several types of microarrays. Then, classifiers were created based on these results to determine the molecular subtype. When developing these classifiers, the application to high-throughput sequencing data, especially single-cell data, was not considered. In this paper, we created OVsignGenes, a neural network model for determining the HGSC subtype that can process bulk RNA-Seq or single-cell RNA-Seq data, including spatial transcriptomic data.

## 1. Introduction

Most cases of epithelial ovarian cancer are represented by high-grade serous carcinoma (HGSC), also known as high-grade ovarian cancer [1]. It accounts for more than 70% of all epithelial ovarian cancers and more than two-thirds (about 70%) of all deaths from ovarian cancer. The main reason for its high mortality is detection at a later stage (stages III and IV), with widespread metastases in the abdominal cavity [2]. It can arise from the ovary, fallopian tubes, or peritoneum and has a worse prognosis than other epithelial cancers [3,4]. Most tumors are immunoreactive to p53 and WT1 proteins and have an increased expression of Ki-67 [5]. The estrogen receptor is expressed in approximately two-thirds of cases [5]. HGSCs vary widely among patients and within individual tumors, with a low overall five-year survival rate of 20–30%. The low survival rate is mainly due to a large tumor burden and extensive metastatic lesions of the peritoneum at the time of diagnosis, which leads to difficulties in achieving complete resection despite advances in surgical techniques [6].

The high degree of heterogeneity in HGSCs can be explained by differences in molecular mechanisms. This is evident from numerous studies on other cancers, including breast cancer, prostate cancer, and lung cancer. Specific molecular subtypes of the above cancers have been identified, which show differences in their progression, recurrence, and overall survival rates. These differences have led to the development of more targeted therapeutic strategies.

Now, attempts have already been made to identify molecular subtypes of ovarian cancer by several scientific groups [7,8,9,10]. The initial study was conducted by Tothill, who divided ovarian cancers into four subgroups based on their molecular and histopathological features [7]. Affymetrix microarrays were used to analyze 285 ovarian tumors [7]. One subgroup (C1) was identified by its reactive stromal signature, which correlated with extensive desmoplastic changes in these samples. Tumors with the C2 signature were characterized by intratumoral infiltration of immune cells, while C4 tumors had relatively low expression of stromal genes and high levels of circulating CA125. The C5 subtype reflected the gene expression signature of mesenchymal cells, and these tumors had rare infiltration of immune cells and were associated with low levels of circulating CA125 [7]. Based on these results, a first classifier based on molecular subtypes (C1, C2, C4, C5) was developed [8].

Then, the Cancer Genome Atlas (TCGA) Consortium measured expression for 11,864 genes using three different platforms: Agilent, Affymetrix HuEx, and Affymetrix U133A. They described four clusters, and a comparison of Tothill’s and TCGA’s clusters showed clear differences that allowed them to conclude there are four reliable subtypes of gene expression. In this work, subtypes were first given names based on their gene clusters: differentiated, immunoreactive, mesenchymal, and proliferative [9]. However, survival was not statistically significantly different for TCGA subtypes in 489 tumor samples studied [9]. Also, another study using gene expression data from microarrays confirmed these subtypes using the consensus clustering method [10]. The difference in survival times between these subtypes was shown here. The longest survival was observed in the immunoreactive subtype, followed by the differentiated and proliferative subtypes, and the worst survival in the mesenchymal subtype [10]. The Ovarian Tumor Tissue Analysis Consortium (OTTA) combined molecular subtypes from previous studies [7,9] into four categories (C1. MES, C2. IMM, C4. DIF, and C5. PRO) using a set of NanoString probes [11]. Another study showed that the mesenchymal subtype had increased expression of myofibroblast/extracellular matrix (ECM) remodeling genes [12,13], and it was more often associated with the presence of metastases in the upper abdominal cavity/omentum [14,15]. Another work based on the assessment of biological pathways showed that the overlap of gene signature estimates suggested that these subtypes were not mutually exclusive [16] and that a tumor could be represented by several dynamic signatures [17].

Understanding the distinctive features of these four subtypes of HGSC, revealing the features of their molecular mechanisms, as well as their high-precision definition, is necessary for the development of modern, effective, and personalized treatment methods. All of the above approaches have been implemented using expression data from microarrays. Transferring these classifiers to HTS data is difficult because possible distortions in their results due to different batches and platforms have not been considered. Currently, the most extensive expression data is obtained by bulk RNA-seq or single-cell RNA-seq, including spatial transcriptomics.

In this work, we focused on identifying stable gene expression signatures for molecular subtypes of HGSC that could be applied to HTS data, as well as on the functional characteristics of these gene modules. We also created and trained a neural network model using single-cell RNA-seq and spatial transcriptomics data to predict the distribution of these signatures.

## 2. Materials and Methods

### 2.1. Cohorts

In total, six datasets were included in this work (Table 1). Cohort_1—high-throughput sequencing data from the TCGA-OV project consisting of 413 patients with labeled data according to four subtypes: differentiated (*n* = 108, 26%), immunoreactive (*n* = 91, 22%), mesenchymal (*n* = 98, 24%), and proliferative (*n* = 116, 28%). Cohort_2—RNA-seq data from ovarian cancer samples from the CPTAC project. Cohort_3—single-cell RNA-seq data of five HGSC samples from PTRC HGSOC project. Cohort_4—three paired samples for which 10x Genomics Visium spatial transcriptomics data is available. Cohort_5—eight samples for which 10x Genomics Visium spatial transcriptomics data is also available. And Cohort_6—single-cell RNA-seq of 41 HGSOCs.

### 2.2. Methods

Differential expression analysis was performed in the R environment (v.3.6.3) [18] using the edgeR package (v.3.24.3, NSW, Australia) [19]. All expression data were presented in the form of counts and normalized to the library size using the trimmed mean of M-values (TMM) method, which calculated counts per million (CPM), considering normalization coefficients. The quasi-likelihood F-test (QLF) and Mann–Whitney U-test (MW) were used to evaluate the significance of changes in gene expression. Benjamini–Hochberg correction was applied to calculate the FDR for all tests. Gene passing *p*-value QLF or MW ≤ 0.05 were considered differentially expressed. Gene set variations (GSVA) were analyzed using GSVA packages (v.1.34.0, Barcelona, Spain) [20] and the Kyoto Encyclopedia of Genes and Genomes database (KEGG, Kyoto, Japan) [21].

Predictive models were created using the machine learning method, neural networks. To build a fully connected neural network (FCNN), the keras [22] and the tensorflow [23] libraries were used. Before creating and training the model, Cohort_1 was divided into training and testing datasets (2:1), scaled and centered using the scale R function. The architecture of the model is a deep network containing 10 hidden layers. Sets of predictors (*n* = 357) were used as the input layer. Each hidden layer consisted of 50–750 neurons with a “swish” activation function. The output layer was four neurons according to four subtypes with the activation function “softmax”. The “categorical_crossentropy” was used as the error function. The algorithm “adam” with the parameter learning rate = 0.003 was used as an optimizer [24]. To prevent overfitting, the model was subject to preservation after passing each epoch. The number of epochs giving the maximum quality of the model was considered optimal (maximum number of epochs = 500). The metrics of model quality during training were normalized proportion of correct answers (Cohen’s kappa), accuracy, precision, sensitivity, specificity, and area under the error curve (AUC). The constructed models were subject to ROC analysis (receiver operating characteristic, R package pROC, v.1.18.0) [25]. Thus, four primary models were created on each of the gene sets (according to four control genes). Then, these models were combined into a single architecture with four parallel channels, the estimates of which are averaged by means of an additional layer. The model metrics were calculated using the caret R package (v.6.0-93) [26].

Visualizations of the obtained results were performed using the ggplot2 package (v.3.4.2) [27].

## 3. Results

### 3.1. Gene Expression Signature of the Four Molecular Subtypes

**Figure 1:**
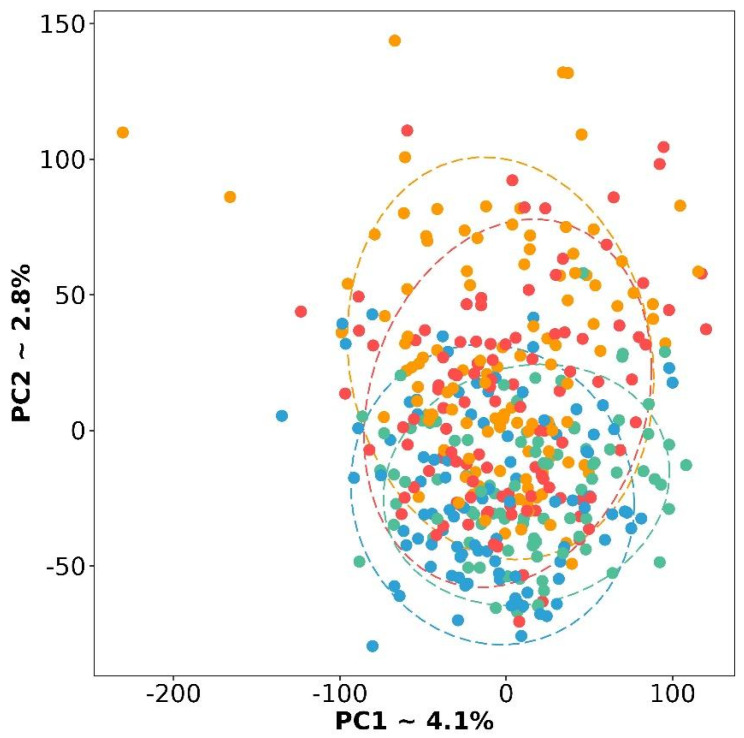
PCA plot based on gene expression for four molecular subtypes of HGSC. Red color is a differentiated subtype, green—immunoreactive, blue—mesenchymal, and yellow—proliferative.

Previously, four molecular subtypes were described: differentiated (D), immunoreactive (I), mesenchymal (M), and proliferative (P) [9]. As a first step of the study, we verified that these molecular subtypes have expression variability on RNA-seq data. To do this, we performed an analysis of the principal components (PCA) and displayed the results in Figure 1. It should be noted that the subtypes really differ from each other, although the differentiated subtype is located at the junction of the other three.

The next step was to analyze the differential expression (DE) in several designs, namely, four comparisons, when one subtype is opposed to the other three: (1) D vs. other, (2) I vs. other, (3) M vs. other, (4) P vs. other, as well as six pairwise comparisons, (5) D vs. I, (6) D vs. M, (7) D vs. P, (8) I vs. M, (9) I vs. P, and (10) M vs. P. Detailed results of all comparisons are presented in additional Supplemental Table S1. It was advisable to make pairwise comparisons since designs 1–4, when one subtype is opposed to the other three, gave extremely few DE genes for the D subtype (only four). Perhaps this is because combining the three subtypes does not provide the necessary homogeneous group for opposition. Whereas, pairwise comparisons yielded several hundred DE genes (Supplemental Table S2).

**Figure 2:**
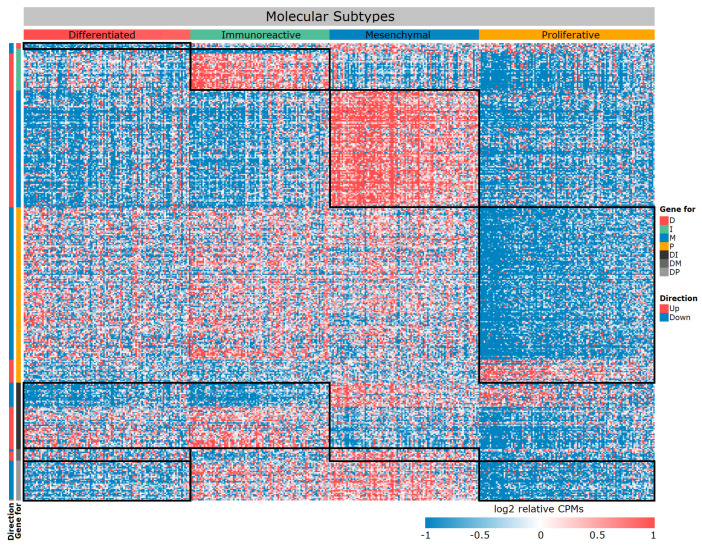
Heat map of differentially expressed genes between four molecular subtypes. The black frame—specific gene module, rows—genes, columns—samples.

We crossed these lists and obtained 1390 unique DE genes between subtypes. Because we initially assumed to provide a multiplatform for our approach, we have left only those genes from this list that were also expressed in samples from datasets 2–6 (Table 1). Thus, we have formed a list of 357 DE genes, which we have marked up according to the direction of expression change in four subtypes (Table 2, Figure 2, Supplemental Table S3).

**Figure 3:**
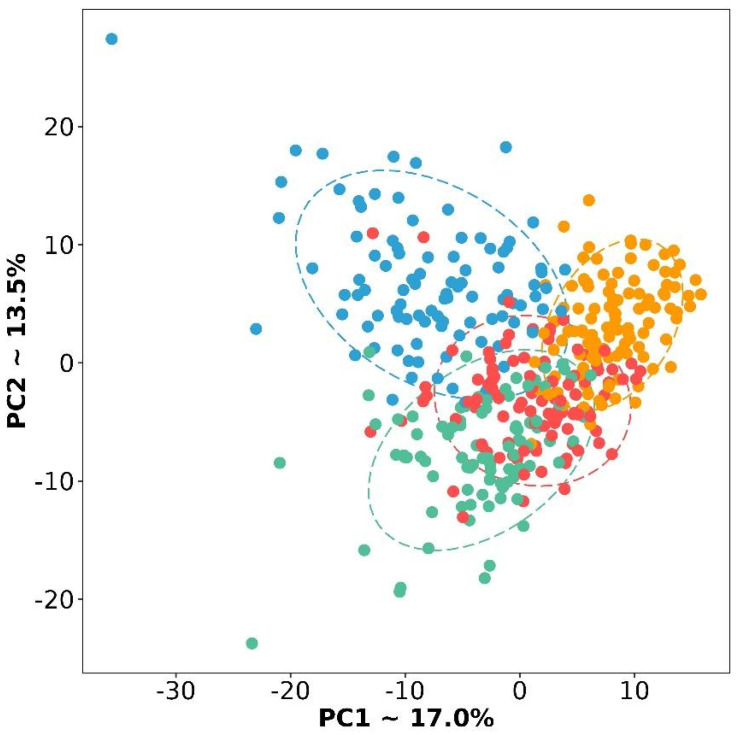
PCA plot of four subtypes based on selected 357 DE genes. Red color is a differentiated subtype, green—immunoreactive, blue—mesenchymal, and yellow—proliferative.

If you carefully look at Table 2 and Figure 2, you will notice very few genes specific to the D subtype. We repeated the PCA based only on 357 selected DE genes (Figure 3). Subtypes form more separate groups, but the D subtype still lies at the junction between the other three. With respect to their expression profiles, it is similar to all other subtypes, but only a specific gene set can distinguish it from the other two subtypes (Figure 2 and Figure 3). In particular, a number of genes exhibit extremely similar expression patterns, for example, in subtype D and I, but they differ in M and P. Therefore, this subtype can be separated from the others by pairwise exclusion based on the identified genes.

In addition, to control the effects of platforms and individual batches, we selected control genes (*SLC25A39*, *HUWE1*, *ATXN2L*, and *EIF1*) to normalize DE genes within each sample. The control gene should not be DE (absolute Log2FC < 0.2 and *p*-value ≥ 0.05); it should have an expression of at least 7 Log2CPM.

### 3.2. Functional Characteristics of Differentially Expressed Genes

**Figure 4:**
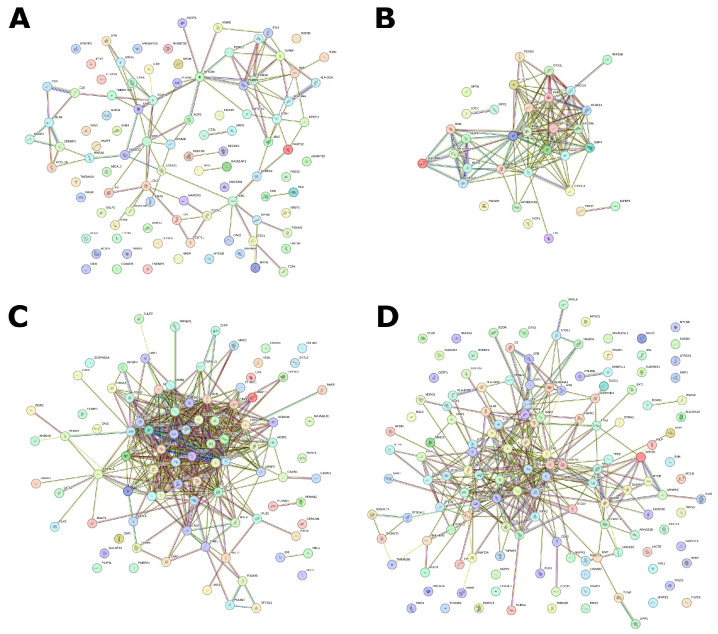
STRING network based on special DE genes for each of four subtypes. (**A**) D subtype, (**B**) I subtype, (**C**) M subtype, and (**D**) P subtype.

Of particular interest is the annotation of these gene sets based on KEGG biological pathways. Based on an analysis of differential gene expression, we constructed a STRING network and identified enriched biological pathways in each of the four molecular subtypes (Figure 4, Supplemental Table S4).

For I subtype 34, upstream regulated enriched pathways were identified (Supplemental Table S5). The top five include allograft rejection (hsa05330), graft versus host disease (hsa05332), type I diabetes mellitus (hsa04940), Th17 cell differentiation (hsa04659), and antigen processing and presentation (hsa04612). In general, the analysis demonstrates upstream enrichment of processes related to the histocompatibility complex, antigen presentation, and graft rejection reactions. Perhaps this is due to the immune system’s attempt to react to tumor cells.

For M subtype, 26 upregulated enriched pathways were identified (Supplemental Table S5). The top five include ECM-receptor interaction (hsa04512), AGE-RAGE signaling pathway in diabetic complications (hsa04933), protein digestion and absorption (hsa04974), glycosaminoglycan biosynthesis—chondroitin sulfate/dermatan sulfate (hsa00532), and focal adhesion (hsa04510). Here, we can observe the upstream regulated pathways associated with fibril organization, adhesion, and extracellular matrix formation. This may be due to an imbalance in signaling about cellular neighborhoods and a violation of tissue structure, which can lead to invasion and metastasis.

In P subtype, 55 downstream regulated pathways were identified (Supplemental Table S5). The top-five pathways are toll-like receptor signaling pathway (hsa04620) and NOD-like receptor signaling (hsa04621), NF-kappa B signaling (hsa04064), TNF signaling (hsa04668), and complement and coagulation cascades (hsa04610). For the P subtype, the greatest enrichment can be noted in pathways associated with the complement system, calcium binding, and regulation of DNA-binding proteins. Most of these enriched pathways can affect cell proliferation as well as inflammation and apoptosis.

This subtype is the most difficult because the molecular subtype D of ovarian cancer does not have its own clear gene expression pattern. However, it can be identified through analysis in the context of other subtypes. Here, it is more expedient to compare it with other subtypes and collectively analyze the results. The STRING network shows that these genes, which differ in expression in this subtype, do not form any specific interactions, although, of course, it is possible to identify some enriched pathways here (Supplemental Table S5). The top-five pathways in the D subtype are upstream enrichment for biotin metabolism (hsa00780), downstream enrichment for primary immunodeficiency (hsa05340), cell adhesion molecules (hsa04514), Rap1 signaling pathway (hsa04015), and cytokine–cytokine receptor interaction (hsa04060). Basically, this indicates reduced activity of cellular processes related to neighborhood, adhesion, and immune response. This looks much more disjointed compared to the situations with other subtypes, but at the same time, it is similar to individual results for other subtypes in terms of genes and pathways.

### 3.3. HGSC Molecular Subtype Neural Network Model

**Figure 5:**
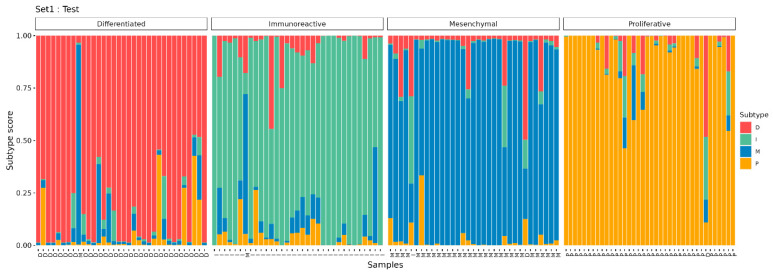
The results of the prediction of molecular subtypes by the model for the test dataset.

Based on the selected 357 DE genes and 4 control genes, OVsignGenes, a four-channel model of a fully connected neural network, was created and trained to determine one of the four molecular subtypes of ovarian cancer. The accuracy of the model was 99% for training data (*n* = 275) and 95% for verification data (*n* = 138; Table 3, Figure 5).

It should be noted that some samples have a mixed signature. For example, when most of the signal is assigned to subtype I, at the same time, the signature of subtype D is also present to a lesser extent (Figure 5). Probably, such samples include areas of the tumor with different signatures and may belong to different subtypes, which is reflected in bulk RNA-seq samples as a proportion of subtype signatures.

### 3.4. Verification of the OVsignGenes Model on External Datasets

To test the operation of the model, we have involved five more different datasets (Table 1). All of them were presented by the authors in the form of gene expression counts, which were processed and normalized as described above. The independent Cohort_2 represented 62 samples, for which bulk RNA-seq was also performed, as well as for Cohort_1. According to the estimates of the model, two samples belonged to the D subtype, 23—I, 35—M, and 2—P.

Cohort_3 contained five samples for which scRNA-seq was performed. Here, the model annotated individual cells according to their expression profile. The estimates of these profiles are then combined into an overall result for each sample. Four of the five samples are assigned to subtype I, and only one is assigned to M.

**Figure 6:**
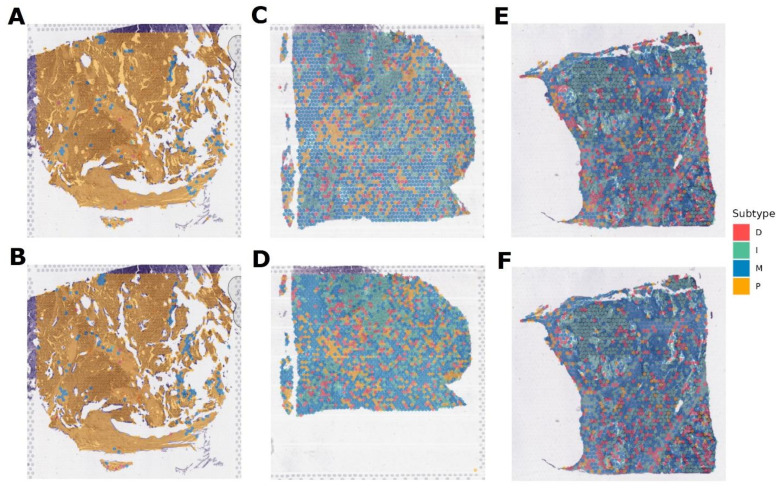
Molecular subtypes of Cohort_4 samples for which gene expression was obtained by spatial transcriptomics. (**A**,**B**) pair are replicas of the same tumor slide belonging to the P subtype. (**C**,**D**) pair are replicas of one tumor slide, belonging mainly to the M subtype. (**E**,**F**) pair are replicas of one tumor slide, belonging mainly to the I and M subtypes.Cohort_5 contained eight samples, for which spatial transcriptomics was also performed (Figure 7). The subtype M was determined for four samples (Figure 7A–C,E) and P for three of the eight samples (Figure 7D,F,H). However, for sample D, it should be noted that in addition to the subtype P, there is an area of subtype M. And one sample (Figure 7G) is assigned to the D subtype, but with a significant interspersing of the P signal.

Cohort_4 contained three paired samples (six in total), for which spatial transcriptomics was performed using two different protocols (Figure 6). In this case, the model evaluated small clusters of nearby cells according to the resolution of the sequencing method. As a result, for the Figure 6A,B pair, the subtype is defined as P. For pair Figure 6C,D—M subtype, however, it can be noted that in this sample, there is also a region for which the I subtype is defined. And for the Figure 6E,F pair, the result is not unambiguous. Specifically, for Figure 6E, 38% of the area fell on subtype I and 35% on M. For Figure 6F, it is a little different: 34% is I, and 44% is M subtype. Probably, this sample is represented by two subtypes at once in approximately equal ratio. For this pair, there are also regions with a D signature, but there are much fewer of them.

Cohort_6 included 41 samples for which scRNA-seq was performed. The processing was performed by analogy to Cohort_3. Of all the samples, 11 were assigned to the D subtype, 5—I, and 25—P.

Thus, we determined the subtypes using the created model on another 122 samples, which included data obtained by bulk RNA-seq, scRNA-seq, and spatial transcriptomics. It should be noted that running the model through expression data from different platforms shows that a single sample can indeed have several signatures corresponding to different subtypes. However, this is mostly local in nature: there are areas in the tumor where the tissue architecture corresponds to one subtype, while another part of the same tumor has inclusions of a different subtype.

## 4. Discussion

Previously, based on cluster analysis of gene expression from microarray data, the TCGA Consortium described four molecular subtypes: differentiated (D), immunoreactive (I), mesenchymal (M), and proliferative (P) [9]. These TCGA subtypes had a fairly high level of concordance with other methods, although they did not always coincide in absolute terms [7,8,10,11].

In current work, we first transferred the study to HTS level and identified 357 DE genes that clearly separated these four subtypes. Based on these genes, a neural network model was created with control genes used to normalize and eliminate the effects of platforms. The model was applied to define subtypes in five external datasets, including bulk RNA-seq, scRNA-seq, and spatial transcriptomics.

First, it is important to note the special position of the D subtype. Throughout the work, this subtype looks more ambiguous than all others. On PCA charts, it is located at the intersection of the other three subtypes. When doing DE analysis in the mode “1 subtype vs. the rest”, there are very few DE genes for the D subtype, but when doing pairwise analysis, much more DE genes can be identified. Perhaps this is because combining the three subtypes does not provide the necessary homogeneous group for opposition. No work based on microarrays mentions such features of the D subtype. The GSVA in the KEGG biological pathways gives an ambiguous picture of this subtype as well. Essentially, this subtype has specific features of the other three subtypes. This allows it to be identified only by exclusion, whereas the other subtypes have a clear and unambiguous status.

In general, I and M subtypes are characterized primarily by increased expression of specific gene blocks and upstream regulation of pathways. In contrast, P subtype is characterized by loss of expression of several genes and corresponding downstream pathway regulation. Both manners of changes are present in D subtype, but they are not unique to it, i.e., they are mostly present in one of three subtypes. Similar results were obtained by TCGA on microarrays. According to its conclusions, the D subtype had a less clear picture, while I/M subtypes had pronounced upregulation of gene expression blocks. P subtype, on the other hand, was characterized by decreased expression of genes [9]. In addition, recent studies based on the analysis of biological pathways have reported that these subtypes are not mutually exclusive [16] and that a single tumor can be represented by several signatures corresponding to several subtypes [17].

It should be noted that running the model through expression data from different platforms shows that a single sample can indeed have several signatures corresponding to different subtypes. However, this is mostly local in nature: there are areas in the tumor where the tissue architecture corresponds to one subtype, while another part of the same tumor has inclusions of a different subtype.

At the moment, there is another study in which they tried to evaluate the subtype using the consensusOV subtype classifier for five samples, for which scRNA-seq was performed (in our study, it is Cohort_3 [28,29]). It was determined that more than 70% of tumor cells in each of these samples belong to subtype D, with a small admixture of other subtypes [30]. However, our approach gave different results for the same samples (I and M subtypes).

Considering that subtype D occupies a position at the junction of the other three subtypes, it does not have its own unique profile of DE genes or enriched pathways. Also, considering the fact that the subtypes are not mutually exclusive and can be dynamic [30], it seems that subtype D may be an initiating subtype, i.e., the first chronological subtype. This point of view has been previously indicated in the work described above [30]. However, there is also an assumption that this subtype may further develop into one of the other subtypes within the framework of tumor evolution. However, further research is needed to confirm this.

As a limitation of the method, it should be noted that it is difficult to implement into clinical practice on a large scale. It is well known that bulk RNA-seq is an expensive procedure and not available to every clinic (with a flow of patients) or individual patient.

## 5. Conclusions

In our work, we considered the possibility of classifying HGSC into molecular subtypes based on expression data obtained by high-throughput sequencing. We reviewed both bulk RNA-Seq and scRNA-seq, as well as spatial transcriptomics data. We identified 357 differentially expressed genes and created a model based on their relative expression levels. The OVsignGenes neural network model was able to process data from these methods and evaluate membership in one of the four subtypes. We confirmed that signatures of several subtypes can be present within the same tumor, and a heterogeneous signature was identified for the D subtype, which had some similarities to the other subtypes but required further detailed study. This subtype may be an initiating subtype unlike the others.

## Figures and Tables

**Figure 7:**
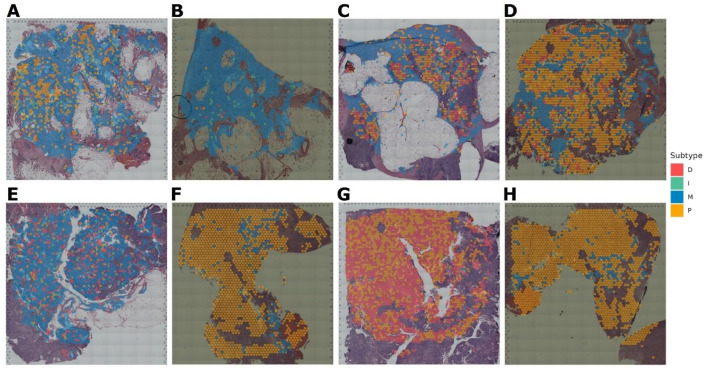
Molecular subtypes of Cohort_5 samples for which gene expression was obtained by spatial transcriptomics. (**A**–**C**,**E**) slides belong to M subtype. (**D**,**F**,**H**) slides—P subtype. (**G**) slide belong mainly to D subtype.

**Table 1:** Cohorts used in the work.

Cohort	Description	Cases, *n*	PID
1	TCGA-OV, Bulk RNA-seq	413	21720365
2	CPTAC, Bulk RNA-seq	62	25873244
3	PTRC-HGSOC, scRNA-seq	5	37541199
4	Spatial ovarian cancer 6, 10x Genomics Visium spatial transcriptomics	6	36882687
5	Spatial ovarian cancer 8, 10x Genomics Visium spatial transcriptomics	8	36788074
6	Ovarian cancer, scRNA-seq	41	36517593

**Table 2:** Number of DE genes for each subtype.

Genes, *n*	D				I	M	P
	D vs. Other	DI vs. MP	DM vs. IP	DP vs. IM			
Up	0	33	7	1	29	91	18
Down	4	19	2	30	4	0	119
Total	96				33	91	137

**Table 3:** Metrics of the quality of the OVsignGenes model for each subtype on the test dataset.

Subtype	Sensitivity	Specificity	Precision	Accuracy	Kappa	AUC
D	0.98	0.97	0.99	0.98	0.94	0.976
I	0.99	0.97	0.99	0.99	0.96	0.980
M	0.98	0.94	0.98	0.96	0.92	0.960
P	0.99	0.97	0.99	0.99	0.98	0.987
All	0.96	0.99	0.96	0.98	0.95	0.969

## Data Availability

The data used in the article are publicly available and links to them are provided in the Cohorts section in Table 1.

## Supplementary Materials

The following supporting information can be downloaded at: https://www.mdpi.com/article/10.3390/cancers16233951/s1, Table S1: differential expression results; Table S2: passed differentially expressed genes; Table S3: selected genes for model; Table S4: pathway enrichment results; Table S5: passed enriched pathways.
